# Next Generation CAR T Cells for the Immunotherapy of High-Grade Glioma

**DOI:** 10.3389/fonc.2019.00069

**Published:** 2019-02-26

**Authors:** Christopher T. Petersen, Giedre Krenciute

**Affiliations:** Department of Bone Marrow Transplantation and Cellular Therapy, St. Jude Children's Research Hospital, Memphis, TN, United States

**Keywords:** CAR T cells, glioblastoma, adoptive cell transfer (ACT), cell engineering, gene editing crispr

## Abstract

High grade gliomas (HGG) comprise a heterogeneous group of brain malignancies with dismal prognosis. Current standard-of-care includes radiation, chemotherapy, and surgical resection when possible. Despite advances in each of these treatment modalities, survival rates for pediatric and adult HGG patients has remained largely unchanged over the course of several years. This is in stark contrast to the significant survival increases seen recently for a variety of hematological and other solid malignancies. The introduction and widespread use of immunotherapies have contributed significantly to these survival increases, and as such these therapies have been explored for use in the treatment of HGG. In particular, chimeric antigen receptor (CAR) T cell therapy has shown promise in clinical trials in HGG patients. However, unlike the tremendous success CAR T cell therapy has seen in B cell leukemia and lymphoma treatment, the success in HGG patients has been modest at best. This is largely due to the unique tumor microenvironment in the central nervous system, difficulty in accessing the tumor site, and heterogeneity in target antigen expression. The results of these features are poor CAR T cell proliferation, poor persistence, suboptimal cytokine secretion, and the emergence of antigen-loss tumor variants. These issues have called for the development of “next generation” CAR T cells designed to circumvent the barriers that have limited the success of current CAR T cell technologies in HGG treatment. Rapid advancements in gene editing technologies have provided several avenues for CAR T cell modification to enhance their efficacy. Among these are cytokine overexpression, gene knock-out and knock-in, targeting of multiple antigens simultaneously, and precise control of CAR expression and signaling. These “next generation” CAR T cells have shown promising results in pre-clinical models and may be the key to harnessing the full potential of CAR T cells in the treatment of HGG.

## Introduction

**Figure 1:**
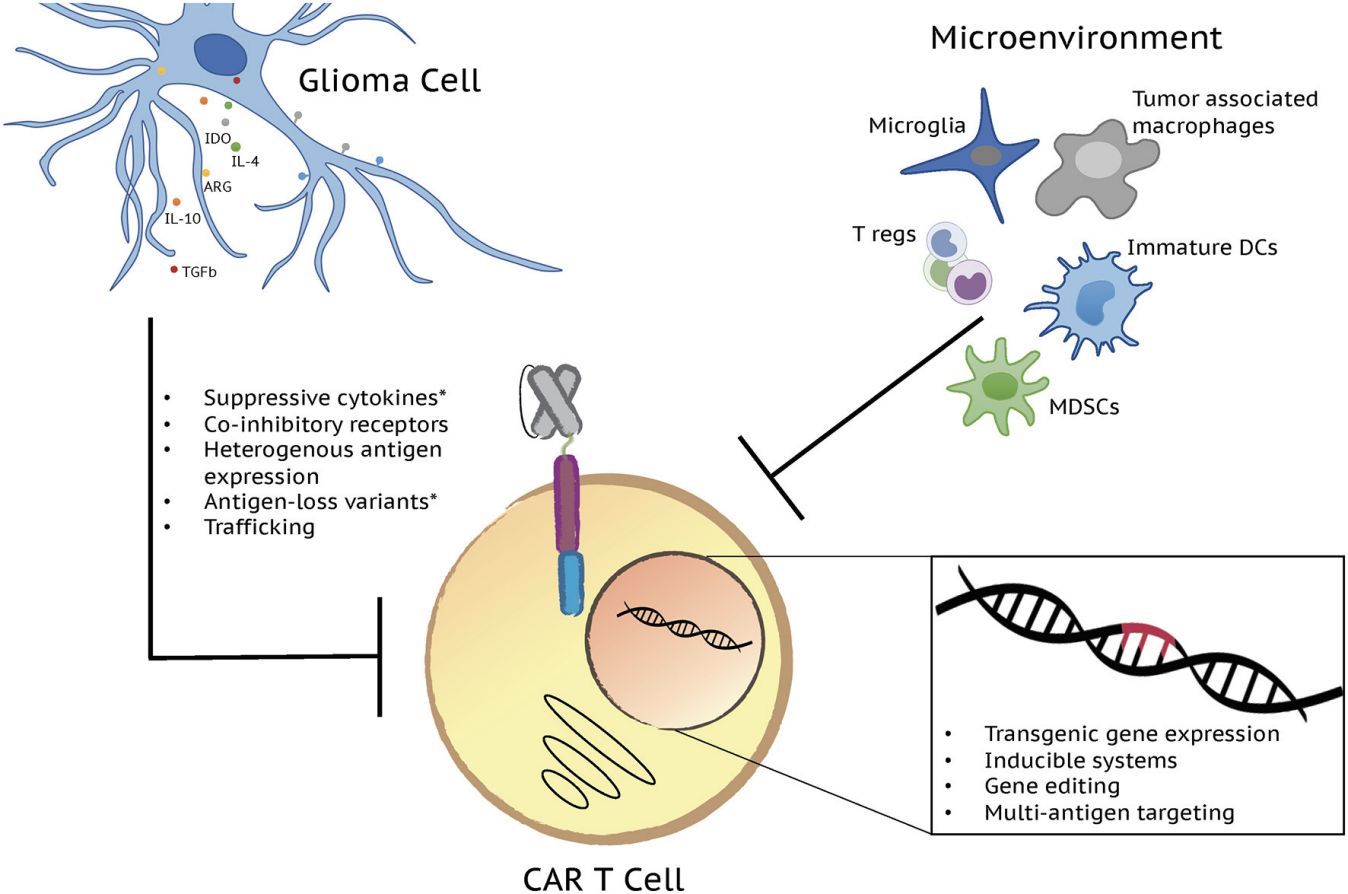
Next generation CAR T cells have potential to overcome factors influencing limited CAR T cell effector function in gliomas. A variety of both T cell and tumor-intrinsic factors add to the lack of efficacy in brain tumors. Limited T cell persistence, exhaustion, poor trafficking, and a hostile tumor microenvironment, defined by immunosuppressive cell populations and molecules, as well as antigen escape contribute to CAR T cell dysfunction and disappointing clinical results. Each of these challenges is being addressed by the development of the next generation of CAR T cell therapies through transgenic gene expression, inducible systems, gene editing, and multi-antigen targeting. Major immunosuppressive cell populations and molecules along with the genetic strategies currently being tested to overcome them are depicted in the figure. *Challenges/issues reported from clinical trial results.

The use of adoptively-transferred T cells as an anti-cancer therapeutic is a concept that has been explored extensively over the past several years. Early attempts at targeting tumors for T cell-mediated destruction largely focused on the use of vaccinations and the adoptive transfer of tumor infiltrating lymphocytes (1). Despite promising pre-clinical results, these approaches proved to be of limited clinical utility necessitating the development of alternative approaches. Modern advances in molecular genetic and cloning technologies enabled the development of T cells in which tumor targeting could be precisely controlled by the expression of a chimeric molecule containing domains for both antigen specificity and activation. Canonical T cell signaling involves binding of the T cell receptor (TCR) to a peptide antigen in complex with a major histocompatibility complex molecule and downstream signaling through the CD3 complex and co-stimulatory receptors (2). Chimeric antigen receptors (CARs) mimic this pathway through inclusion of either a single chain variable fragment (scFv) derived from a monoclonal antibody or a mutated ligand for target binding, one or more co-stimulatory domains, and the ξ chain of the CD3 complex all in a single multi-domain receptor (3). Introduction of a CAR into T cells endows them with the ability to kill cells expressing a target antigen through the same effector functions used by wild type T cells to eliminate infected or transformed cells (3). CAR T cells have revolutionized the field of tumor immunotherapy following tremendous success in the treatment of B cell malignancies. Building off of these clinical successes, the use of CAR T cells has also been explored in a variety of solid malignancies including high grade gliomas. In contrast to their efficacy in B cell malignancies, however, CAR T cells have to date shown limited activity in solid tumor settings (4). A variety of both T cell and tumor-intrinsic factors contribute to this lack of efficacy in solid tumors as well as in patients with B cell malignancies who fail to respond to CAR T treatment. Limited T cell persistence, exhaustion, poor trafficking, and a hostile tumor microenvironment as well as antigen escape contribute to CAR T dysfunction (Figure 1) and are each being addressed in the development of the next generation of CAR T therapies.

Genetic modifications of CAR T cells have demonstrated to have significant effects on their function and efficacy as initially evidenced by the insertion of co-stimulatory domains into the first-generation construct (5, 6). The development of highly efficient lentiviral and retroviral vectors has enabled the insertion of larger constructs containing multiple genes into the T cell genome. In addition to modification of CAR constructs and viral vector-mediated random insertion of additional genes, newer strategies take advantage of more targeted gene editing technologies such as TALEN and CRISPR/Cas systems to modify the T cell genome. Such technologies enable the knock-out of negative T cell regulators through targeted gene disruption as well as the knock-in of transgenes. Excitingly, recent advances in gene editing techniques have enabled highly efficient homology-directed repair in T cells for combined gene knock-out and knock-in approaches (7). Each of these technologies has contributed to the development of next generation gene-modified CAR T cells that have shown significant improvements over the current generation of CAR T cells being used clinically. While the majority of pre-clinical studies using these CAR T cells have been performed in models of B cell malignancies and solid tumors, the same strategies can be employed to improve CAR T cells for use in high grade gliomas. These malignancies have long posed significant clinical challenges and have demonstrated to be highly resistant to current CAR T therapies. As such, next generation gene-modified CAR T cells are an attractive approach to overcome both the T cell and tumor-intrinsic factors that have resulted in poor CAR T efficacy in high grade gliomas. The strategies to be discussed are summarized in Table 1. The next-generation approaches to improve CAR T cells include transgenic gene expression, gene editing, multi-antigen targeting and inducible systems. While there are many ways to engineer CAR T cells and enhance their effector function, we will only review strategies that have been tested in brain tumor models or those that have the highest potential to address challenges posed by brain tumor biology and/or the tumor microenvironment.

**Table 1:** Summary of the next generation approaches to improve CAR T cell efficacy.

**Strategy**	**Genes/targets/systems**	**Tested in glioma?**	**Results**	**References**
Cytokine overexpression	IL-12	No	Improved anti-tumor activity and persistence	(8, 9)
	IL-15	Yes	Improved anti-tumor activity and persistence, antigen escape	(10)
	IL-18	No	Improved anti-tumor activity and persistence	(11, 12)
Cytokine receptor (constitutively active)	IL-7R	Yes	Improved anti-tumor activity and persistence	(13)
Knock-out	PD-1	No	Improved anti-tumor activity	(14–16)
	DGK	Yes		(17, 18)
Knock-in	TRAC	No	Improved CAR expression and anti-tumor activity	(7, 19)
	CXCR4	No	Proof of concept of successful CRISPR/Cas9-mediated HDR in human T cells	(20)
	PD-1	No		(20)
Multi-antigen targeting	HER2+IL13Rα2	Yes	Improved anti-tumor activity, antigen escape prevention	(21)
	HER2+IL13Rα2+EphA2	Yes		(22)
Controlled and inducible systems	Syn/Notch	No	Controlled CAR expression	(23)
	Inducible co-stimulation	No	Inducible CAR activation	(24)

## Transgenic Gene Expression: Cytokine Overexpression

In addition to second and third generation CAR T cells, fourth generation CAR T cells have been generated that incorporate a third stimulatory signal. Sometimes referred to as “TRUCK” T cells, fourth generation CAR T cells are defined as CAR T cells armed with immune stimulatory cytokines (25) that improve CAR T cell expansion and persistence while rendering them resistant to the immunosuppressive tumor environment. Additionally, transgenic cytokine expression can potentially trigger bystander T cells to eliminate antigen-negative cancer cells at the target site. Cytokines that have been studied using this approach include IL-12, IL-15, and IL-18 (8, 9, 11, 12, 26). IL-12- and IL-18-producing CAR T cells have been tested in both hematological and solid malignancies. In regards to high grade glioma, IL-15 has been tested in a xenograft model of glioblastoma (10). IL-15 belongs to the common γ-chain cytokine family and has activities important for T cell expansion and survival (27). It also has structural similarity to IL-2, but unlike this cytokine, IL-15 does not promote the development of regulatory T cells but rather supports the development of T-memory stem cells (Tscm) which have been demonstrated to have superior *in vivo* function and persistence (28, 29). Moreover, increased *IL-15* gene expression in the tumor microenvironment correlates with improved survival of colorectal cancer patients (30). This indicates that IL-15 has great potential to improve the function of CAR T cells.

In glioblastoma studies, CAR T cells targeting IL-13Rα2 were modified to over-express transgenic IL-15 and demonstrated that IL-15 cytokine secretion was T cell activation dependent and resulted in improved CAR T cell persistence *in vitro*. This also translated into significantly improved anti-tumor activity *in vivo*. However, late recurrences were observed after treatment with CAR T cells producing transgenic IL-15 (10). Despite the observed tumor relapse, this study showed that genetic modification of CAR T cells, in this particular case—cytokine overexpression, can be a powerful method to greatly improve CAR T cell function and persistence. Complete tumor eradication may require a multi-antigen targeting approach.

The question still remains if the soluble form of IL-15 is the best way to express it within CAR T cells. Studies testing membrane-bound (mb) IL-15, tethered to the membrane through a CD8α transmembrane domain, showed enhanced anti-tumor activity when tested in natural killer (NK) cells (31). Additionally, a study by Hurton et al. tested mbIL-15 tethered to the membrane using full-length IL-15 bound to the IL-15Rα receptor via a flexible linker. This was co-electroporated into T cells with a second generation CD19 CAR construct (26). The rationale for this approach was that IL-15 bound to IL-15Rα closely mimics endogenous trans-presentation of IL-15 which has been shown to enhance T-cell persistence (32). Second generation CD19-specific CAR T cells expressing mbIL-15 showed improved effectiveness *in vitro* and *in vivo* that was attributed to the enrichment of long-lived T-memory stem cell subset (CD45RO-CCR7+CD95+) (26). Mechanistic studies showed that the emergence of Tscm was due to signaling via STAT5. These data show a clear benefit of IL-15 tethered to the membrane. However, such an approach would require modification of T cells by two viral vectors since due to the large size of the transgenes making it difficult to express CAR and mbIL-15 within the same plasmid. The remaining question is if IL-15 is the best cytokine to improve the efficacy of glioblastoma-targeted CAR T cells. IL-12 and IL-18 are the other two γ-chain family cytokines that showed promising results when tested in the settings of hematological malignancies and solid tumors, however, neither has been tested in the brain tumor setting (8, 9, 11, 12). Finally, when overexpressing immune stimulatory cytokines safety must be addressed. Improved safety can be achieved through incorporating suicide genes or safety switches.

Another way to overcome potential toxicity from secreted cytokines is to use a constitutively active cytokine receptor. Such a system will activate cytokine regulated pathways, but it will not be dependent on cytokine availability in the tumor milieu. Investigators characterized constitutively active IL-7 receptor (C7R) co-expressing GD2-specific CAR T cells and showed that this system is capable of improving T-cell proliferation, survival and anti-tumor activity (13). They also co-expressed C7R with a glioma antigen targeting EphA2-CAR in T cells and demonstrated that gliomas were completely eliminated at a cell dose where unmodified EphA2-specific CAR T cells had no activity. However, systems such as C7R do not completely obviate the need for a suicide switch since a constitutively active receptor has the potential of inducing antigen-independent T cell proliferation. It is important to note, however, that the authors of this study did not observe antigen-independent T cell proliferation.

## Gene Editing: Knock-out of Negative T Cell Regulators

The importance of co-stimulatory and co-inhibitory signals in anti-tumor T cell responses has received significant attention in the past decade due in large part to the efficacy of checkpoint blockade in the treatment of solid tumors. In particular, monoclonal antibodies blocking CTLA-4 or PD-1 have seen varying degrees of success in a variety of solid tumors including non-small cell lung cancer (33) and metastatic melanoma (34, 35). Trials utilizing these monoclonal antibodies led to the first FDA-approved checkpoint inhibitor in 2011 and launched investigations into additional targets including TIM3 and LAG3 (36). Although CAR T cells do not signal through the canonical T cell receptor following recognition of their target antigen, the same checkpoint molecules are induced due to the recruitment of similar intracellular signaling molecules (37, 38). As such, checkpoint blockade has been studied as an adjunct to improve the efficacy of CAR T therapy in pre-clinical models including models of glioma (39).

The widespread adoption of gene editing technologies that allow the targeting of specific genes has enabled studies examining the concept of cell-intrinsic checkpoint blockade. In such studies, CAR T cell function is enhanced through the knock-out of negative regulators rather than through the use of adjunct checkpoint blockade therapies. Being a new concept in the field of CAR T therapy, few studies have been published that test this approach. Of the limited numbers of gene targets described to date, the effects of PD-1 knockout has been the most extensively described. One study examined the effects of CRISPR/Cas9-mediated PD-1 disruption in CD19 CAR T cells in a subcutaneous model of CD19+ myelogenous leukemia engineered to overexpress PD-L1. The authors observed enhanced *in vitro* cytotoxicity and significantly reduced tumor burden in mice receiving PD-1 knock-out CD19 CAR T cells compared to control CD19 CAR T cells (14). A related study utilized a CRISPR/Cas9 system to knock-out PD-1 in CD19 and PSCA CAR T cells in xenograft models of B cell leukemia and prostate cancer. In both models, knockout of PD-1 resulted in significantly enhanced anti-tumor activity. Importantly, to highlight the validity of CRISPR/Cas9-based gene editing approaches, the authors noted that the use of CRISPR/Cas9 gene editing did not impair the effector functions of their CAR T cells when compared to non-electroporated cells (15). Another study examined the effect of shRNA-mediated PD-1 knock-out in mesothelin-specific CAR T cells in a xenograft model of mesothelioma. Ablation of PD-1 signaling via shRNA or through the use of dominant-negative PD-1 resulted in significant enhancement of CAR T proliferation, cytokine secretion, and cytotoxicity. Importantly, however, the authors noted that the use of shRNA to knock-out PD-1 did not result in significantly enhanced *in vivo* anti-tumor activity due to low knockdown efficiency (16). Although these studies were not undertaken in models of glioma, their approaches are highly applicable as checkpoint-inhibitor-mediated PD-1 blockade previously has been shown to significantly enhance the effector functions of IL-13Rα2 CAR T cells in animal glioma models (39).

In addition to negative regulators expressed on the surface of T cells, intracellular signaling molecules have also been explored as potential targets for knockdown in CAR T cells. The diacylglycerol kinase DGK has been found to negatively regulate CAR T cell activation through metabolism of diacylglycerol which leads to dampening of Ras and ERK signaling. In one study, murine mesothelin-specific CAR T cells were generated from strains lacking α, ζ, or both α and ζ isoforms. Knock-out of either isoform resulted in significant enhancement of *in vitro* cytotoxicity and IFN-γ secretion with synergistic effects observed in double knockout meso-CAR T cells. Similar effects were observed *in vivo*, with meso-CAR T cells lacking one or both DGK isoforms having significantly increased anti-tumor cytotoxicity compared to wild type CAR T cells (17). A related study was more recently performed examining the effects of DGK knock-out using a CRISPR/Cas9-based strategy in EGFRvIII CAR T cells. Importantly, similar to the previously described study, knock-out of both the α and ζ isoforms demonstrated to have synergistic effects compared to knock-out of either isoform alone. Double knock-out EGFRvIII CAR T cells were significantly less sensitive to TGF-β and PGE2-mediated suppression and did not show significant loss of effector function following repeated stimulation. These *in vitro* findings translated to *in vivo* studies in a mouse glioma model as tumor-bearing mice receiving double knock-out EGFRvIII CAR T cells had significantly reduced tumor burden with increased frequencies of tumor-infiltrating lymphocytes (18). It is important to note, however, that the authors did not use an orthotopic glioma model raising the question of whether this knock-out strategy will be efficacious in the setting of intracranial tumors. Despite this, both of these studies highlight the potential for knock-out of intracellular negative regulators in CAR T cells directed toward glioma antigens.

## Gene Editing: Knock-in of Genes to Enhance CAR T Cell Function

The use of lentiviral and retroviral vectors has enabled the random insertion of a wide variety of transgenes into T cells for several years. Recently, however, targeted insertion has been made possible with a combination of CRISPR/Cas9 and homing nuclease-mediated gene knockout in combination with donor templates delivered by viral and non-viral vectors. The use of these methods has been demonstrated more recently in T cells with a study examining the replacement of the endogenous CXCR4 gene with a mutant variant. The authors used a CRISPR/Cas9-based system to knock-out the endogenous gene while simultaneously providing single-stranded donor template encoding mutant nucleotides. Homology-directed repair of the double strand breaks resulted in high efficiency insertion of the mutant gene leading to reduced surface expression of CXCR4. In addition to CXCR4, the authors used a similar approach to knock-out PD-1 albeit with a donor template that led to a frame shift mutation rather than inserting mutated nucleotides (20). A more recent study utilized CRISPR/Cas9 in combination with donor template DNA to target sequences encoding whole genes into specific T cell loci. The authors demonstrated high efficiency homology-directed repair with high expression of several genes driven by the promoter of the targeted locus. Insertion of a GFP transgene was achieved in multiple loci encoding genes expressed in a variety of cellular compartments. For example, CRISPR/Cas9 knockout of CD4 in combination with donor GFP template led to GFP expression on the plasma membrane while targeting Rab11a led to endosomal GFP expression (40). Both of these studies demonstrate the feasibility of targeted gene editing in human T cells which is an attractive strategy for the enhancement of anti-glioma CAR T cells.

The current methods of retroviral and lentiviral-mediated insertion of CAR constructs rely on random gene integration leading to heterogeneity in CAR expression. To address this, recent studies have aimed at insertion of CAR constructs into the endogenous T cell receptor via targeted gene disruption and homology-directed repair. In one study, a homing endonuclease was used to disrupt the TCR alpha chain locus with donor template being provided by an adeno-associated viral vector. This approach led to highly efficient targeted construct integration and CD19 CAR expression (19). A similar approach was taken in another study using CRISPR/Cas9 to disrupt the TCR alpha chain locus in combination with an AAV6 vector providing the CD19 CAR donor template. Importantly, the authors in this study observed significantly enhanced tumor control and survival in leukemic mice treated with CAR T cells generated by homology-directed repair in comparison to CAR T cells generated by retroviral vectors. The authors found this enhanced efficacy was due to uniformity in CAR expression which resulted in reduced exhaustion following antigenic stimulation (7). Excitingly, a recent study by Fraietta et al. showed that knock-in of a CD19 CAR construct into the *Tet2* locus enhanced the function of anti-CD19 CAR T cells. This was found to be due to random integration of the transgene into the *Tet2* gene which disrupted the endogenous gene's function leading to greater frequencies of central memory T cells (41). Despite the fact that the insertion was random, it can be inferred that targeted knock-in of a CAR construct into the locus of a negative T cell regulator will significantly enhance the therapeutic efficacy of CAR T cells. CRISPR/Cas9 systems would be ideal for this type of approach. Although CAR insertion via the approaches just described has not been tested in glioma models, it can be inferred that such approaches would also be beneficial for the function of anti-glioma CAR T cells as exhaustion has proven to be a significant barrier (42, 43).

## Multi-Antigen Targeting

The initial step toward creating a successful CAR T cell-based therapy is to identify an antigenic target that has minimal off-target recognition potential. Well-defined targets for glioblastoma are IL-13Rα2, HER2, EGFRvIII and EphA2. However, data from pre-clinical and clinical studies have shown that targeting a single antigen leads to down-regulation of the target antigen and subsequent tumor recurrence (10, 44, 45). The mechanism of antigen loss is currently under active investigation as downregulation of the target antigen and/or clonal expansion of antigen-negative tumor cells may contribute to this phenomenon. These findings indicate a need to target multiple antigens to enable CAR T cells to eliminate all brain tumor cells and avoid tumor recurrence. One method to direct CAR T cells to target multiple antigens is by using a dual-antigen targeting CAR that incorporates two antigen targeting domains within one CAR construct. Hegde et al. described a tandem CAR (TanCAR) design which fused a HER2-specific single chain variable fragment (scFv) to an IL-13Rα2-binding IL-13 mutein connected to transmembrane (TM) and signaling domains (21). They then compared anti-tumor activity of TanCAR to bispecific CARs (co-expressed a HER2-CAR and an IL-13Rα2-CAR) and pooled CARs (a pool of uni-specific CARs). They showed that TanCAR was able to effectively lyse glioma cells *in vitro* and showed better anti-tumor activity *in vivo* when compared to single antigen-targeting CAR, bispecific CAR, or pooled CARs. Most importantly, the use of TanCAR resulted in a lack of tumor recurrence.

Later, the same group took a similar approach one step further and designed trivalent CAR T cells targeting 3 glioblastoma associated antigens to overcome patient to patient and tumor cell to tumor cell antigen heterogeneity (22). Using mathematical modeling, the group showed that targeting 3 antigens would address inter- and intra-patient antigenic variability. A trivalent T cell product was designed with HER2, IL-13Rα2- and EphA2-specific CAR molecules all expressed simultaneously on the T cell surface. Tri-specific CAR not only was more efficacious than bispecific CAR but was also more efficient as tumor growth was controlled at lower T cell doses (22).

These proof-of-concept studies present promising and exciting results, however, the question remains if these CAR T cell products will be efficient when tested clinically. In addition, it is unknown if multi-antigen targeting CARTs will be able to sustain their persistence and anti-tumor activity when exposed to the immunosuppressive glioma microenvironment. Due to the potential increased off-target effects when using bi- and tri-specific CAR T cells, the safety of such approaches needs to be evaluated. This can partially be addressed by evaluating and defining more specific targets for glioblastoma. Finally, due to the variation in antigen expression among glioma subtypes, target selection on a patient-by-patient basis may be necessary (46).

## Inducible Systems: Controlling CAR Expression and Activity

It is well established that T cells modified with a single CAR molecule will not effectively eradicate brain tumor cells. As such, second genetic modifications must be introduced to generate more robust and effective CAR T cell products. However, introducing additional modifications brings up safety and toxicity concerns, especially given the unique and sensitive central nervous system environment in which these tumors develop. Thus, when designing new approaches, safety switches, such as suicide genes, or using inducible and/or controllable CAR systems are attractive approaches to address the issue of CAR T cell-induced toxicity.

Inclusion of a suicide gene needs to be considered when targeting tumor associated antigens (TAAs) that are expressed at low levels in normal tissues. Targeting such antigens can result in off-target immune-mediated toxicities. For example, GD2 is a promising target for diffuse intrinsic pontine glioma (DIPG) as CAR T cells targeting GD2 showed strong anti-tumor activity in pre-clinical studies (47). However, it is also well established that GD2 is expressed in normal brain tissues which raises “on-target, off-tumor” toxicity concerns. In fact, severe CNS toxicity has been observed when testing GD2-specific CAR T cells for neuroblastoma with associated T-cell infiltration into brain areas that are known to express GD2 (48). This toxicity can be prevented by inclusion and activation of suicide genes, such as inducible caspase 9 (49). Activation of suicide genes eliminates CAR T cells potentially reducing damage to normal tissues.

Another method to overcome “on-target, off-tumor” toxicity is to use a Syn/Notch system which is based on NOT- and AND-gated CARs. In other words, CAR expression can only be induced upon recognition of a second tumor associated antigen (50, 51). The limitation of such an approach is that both antigens must be expressed at considerably high levels by tumor cells. This requirement poses a potential problem for anti-glioma CAR T cells due to high heterogeneity in antigen expression levels.

One additional option to prevent toxicity and to improve CAR T cell effector function is to control the activity of CAR T cells through inducible systems that provide co-stimulatory signals. In the scenario where a single CAR has limited anti-tumor activity, co-stimulation can be induced through treatment with small molecules. Unlike the most common CAR constructs which produce a single molecule encoding activation and co-stimulatory domains, inducible systems separate the activation and co-stimulatory domains into two distinct molecules. Such a system was described by Mata et al. where they used a combination of HER2-specific CAR and inducible costimulatory molecules that contain a chemical inducer of dimerization (CID)–binding domain and the MyD88/CD40 signaling domains. In the presence of CID, MyD88/CD40 molecules oligomerize and produce co-stimulatory signals to T cells inducing cytokine release and proliferation. This system was able not only to improve and control HER2-specific CAR T-cell activation but also enabled modified T cell reactivation by repeat CID injection *in vivo* (24).

All of the approaches just described demonstrate great potential to improve CAR T cell function and safety, however, the study of these approaches in the setting of high grade glioma is limited at present.

## Conclusions and Future Perspectives

CAR T cell therapies hold immense potential in the treatment of high grade gliomas, malignancies which continue to have dismal outcomes in spite of recent therapeutic advances (52). However, current generation CAR T cells that have been tremendously successful in the treatment of hematological malignancies have had only modest efficacy in glioma patients (4). It is clear that much of the potential of CAR T cell therapy for glioma lies in modification of the cells themselves. Modern genetic technologies such as CRISPR/Cas9 and synthesis of large custom gene constructs enables significant manipulation of CAR T cells to improve their cytotoxicity, persistence, and safety. These methods have proven to be powerful tools in the design of CAR T cells for a variety of malignancies, but their use in studies of glioma is limited at present. In order to determine whether the enhanced anti-tumor activity afforded to CAR T cells through gene editing will also be beneficial in the setting of high grade glioma, these approaches must be tested in preclinical glioma models.

One of the major challenges of designing CAR T cells for glioma treatment is the relatively poor understanding of the central nervous system immune environment (53). Increasing our understanding of the environment that CAR T cells function in is critical to the proper development of manipulation strategies. While modifications that focus on aspects of general T cell biology may prove effective, strategies aimed at addressing the unique biology of glioma and its microenvironment may prove crucial. For example, gene modifications designed to resist the effects of central nervous system-resident myeloid cells may prove especially effective as these cells are potently immunosuppressive (54). Additionally, as evidenced by a recent clinical trial, several immunosuppressive molecules such as indoleamine 2,3-dioxygenase (IDO) are upregulated in gliomas following CAR T cell infusion and contribute to poor T cell persistence (55). Strategies specifically tailored to the unique immunosuppressive environment of the central nervous system may thus be needed in order to develop CAR T cells for brain tumors. Additionally, access to the glioma microenvironment through crossing the blood brain barrier may pose a limitation to CAR T cell therapies. Although CAR T cells have been shown to cross the blood brain barrier following intravenous administration, trafficking of the cells may be suboptimal as evidenced by limited T cell accumulation in the tumors (55). A strategy aimed at manipulating T cell trafficking to the central nervous system has recently been described in a study examining the effect of blocking T cell S1P internalization in murine glioma models (56).

Gene modification approaches that have been developed for hematological and solid malignancies may work well in those settings, but there is a possibility that those same approaches may not work in glioma. As such, glioma-specific factors that inhibit anti-glioma CAR T cells may be identified through genome-wide screening approaches that have recently been described (57, 58). Additionally, the use of immunocompetent mouse models that more faithfully recapitulate the immune and stromal glioma microenvironments will be essential to adequate translation of anti-glioma CAR T cell approaches. This remains a significant issue in the field as the commonly used glioma models are performed in immunodeficient mouse strains which do not accurately model the effects of the tumor microenvironment on CAR T cell responses (59). Importantly, the interaction of CAR T cells with central nervous system-resident myeloid cells.

In summary, the development of genetic approaches to create the next generation of CAR T cells may have a large impact on the immunotherapy of high grade glioma. Multiple strategies such as the insertion of cytokine transgenes, gene knock-out, gene knock-in, control of CAR expression and activity, as well as multi-antigen targeting have tremendous potential in the field of anti-glioma CAR T cell therapy. Choosing the most effective strategy will require a significant increase in preclinical testing. So, while it is unknown which next generation CAR T cell will be most effective, it is clear that there is a lot of work that needs to be done.

## Author Contributions

All authors listed have made a substantial, direct and intellectual contribution to the work, and approved it for publication.

### Conflict of Interest Statement

The authors declare that the research was conducted in the absence of any commercial or financial relationships that could be construed as a potential conflict of interest.
